# Identification and characterization of resistance to cowpea aphid (*Aphis craccivora* Koch) in *Medicago truncatula*

**DOI:** 10.1186/1471-2229-12-101

**Published:** 2012-07-04

**Authors:** Lars G Kamphuis, Lingling Gao, Karam B Singh

**Affiliations:** 1 CSIRO Plant Industry, Private Bag 5, Wembley, WA, 6913, Australia; 2 The UWA Institute of Agriculture, The University of Western Australia, Crawley, WA, 6009, Australia

**Keywords:** Antibiosis, Antixenosis, EPG, Herbivory, Sap-sucking insect, Phloem

## Abstract

**Background:**

Cowpea aphid (CPA; *Aphis craccivora*) is the most important insect pest of cowpea and also causes significant yield losses in other legume crops including alfalfa, beans, chickpea, lentils, lupins and peanuts. In many of these crops there is no natural genetic resistance to this sap-sucking insect or resistance genes have been overcome by newly emerged CPA biotypes.

**Results:**

In this study, we screened a subset of the *Medicago truncatula* core collection of the South Australian Research and Development Institute (SARDI) and identified strong resistance to CPA in a *M. truncatula* accession SA30199, compared to all other *M. truncatula* accessions tested. The biology of resistance to CPA in SA30199 plants was characterised compared to the highly susceptible accession Borung and showed that resistance occurred at the level of the phloem, required an intact plant and involved a combination of antixenosis and antibiosis. Quantitative trait loci (QTL) analysis using a F_2_ population (n = 150) from a cross between SA30199 and Borung revealed that resistance to CPA is controlled in part by a major quantitative trait locus (QTL) on chromosome 2, explaining 39% of the antibiosis resistance.

**Conclusions:**

The identification of strong CPA resistance in *M. truncatula* allows for the identification of key regulators and genes important in this model legume to give effective CPA resistance that may have relevance for other legume crops. The identified locus will also facilitate marker assisted breeding of *M. truncatula* for increased resistance to CPA and potentially other closely related Medicago species such as alfalfa.

## Background

Phloem-feeding aphids are a common pest of important crops world-wide. In temperate regions, approximately a quarter of all plant species are colonized by at least one aphid species [1]. In many cases, the highly specialized mode of aphid feeding causes little apparent, but nevertheless significant damage to the plant and it appears that aphids are able to evade plant defenses while moving their stylets intercellularly, as well as manipulate their host through secretion of saliva into the phloem sieve elements [2,3]. Sap-sucking insects cause damage in susceptible cultivars, directly by modifying plant metabolism and ingesting plant nutrients and in many cases indirectly, for example, through the transmission of plant-pathogenic viruses [4].

Studies using the model plant Arabidopsis have provided valuable contributions to our understanding of basal defense mechanisms against aphid feeding [5-11], whereas the molecular mechanisms underlying resistance gene mediated aphid resistance remain less well understood. The cloning of the *Mi1.2* gene in tomato conferring resistance to potato aphid has given us some insight into *R* gene mediated defense to aphids [12-15], and with studies in soybean [16-18] and the model legume *Medicago truncatula* Gaertn [19-27] a picture is emerging around resistance gene-mediated defense against aphids. For example, these studies have shown the recruitment of specific defense signalling pathways in resistant, but not susceptible plants [15,19,28].

Cowpea aphid (CPA; *Aphis craccivora* Koch) is a serious pest in legume agriculture and has been reported on all continents except the Antarctic. This species has been described as the most important worldwide pest of cowpea, *Vigna unguiculata* L. Walpers, causing significant yield losses when either young seedlings or the pods of adult plants are attacked [29]. Cowpea is a protein-rich legume relied on by over 200 million people in Africa and is highly adapted to poor soils and drought conditions. In recent years, cowpea genetic resources have been developed to aid breeding efforts to improve the crop with a focus on drought tolerance and resistance to diseases, nematodes and pests including cowpea weevil (*Callosobruchus maculates*), thrips (*Thrips tabaci* and *Frankliniella schultzei*), CPA and viruses vectored by CPA [30-34].

CPA also causes serious yield losses in chickpea, a major pulse crop in the Indian sub-continent, where transgenic chickpeas expressing the *Allium sativum* leaf agglutinin (ASAL) gene resulted in a significant reduction in survival and fedundancy of CPA [35]. CPA has also been reported to be a pest on peanuts in Africa, where it vectors several viruses which cause groundnut rosette disease [36] and can also be a serious pest of lentils on the Indian subcontinent [37]. In Australia, CPA has been reported to infest pasture legumes such as subterranean clover (*Trifolium subterraneum* L.), common burr medic (*Medicago polymorpha* L.), alfalfa (*M. sativa* L.), barrel medic (*M. truncatula* Gaertn.) as well as Australia’s major grain legume, narrow leaf lupin (*Lupinus angustifolius* L.); [38,39]. In the US, this aphid species has become a more serious pest of alfalfa in the last decade where high population densities of CPA in alfalfa have been reported in over 20 states [40]. Apart from resistance to CPA in lupins [38] and several resistance loci in cowpea, most of which have been overcome by newly emerged CPA biotypes [29,41], natural resistance to CPA has not been identified in other cultivated legumes including *Medicago* species. [20,37,39].

The most common aphid species recorded on legumes in Australia include: bluegreen aphid (BGA; *Acyrthosiphon kondoi* Shinji), pea aphid (PA; *Acyrthosiphon pisum* Harris), spotted alfalfa aphid (SAA; *Therioaphis trifolii* f. *maculata* Buckton), spotted clover aphid (SCA; *Therioaphis trifolii* Monell), green peach aphid (GPA; *Myzus persicae* Sulzer) and cowpea aphid (CPA; *A. craccivora*). In recent years resistance to most of these aphid species has been identified and characterised in the model legume *Medicago truncatula* Gaertn [21-24] with the exception of CPA resistance. Resistance to BGA, PA and SAA in *M. truncatula* are controlled by single dominant genes and were identified in a pair of closely related lines (Jester and Jemalong A17) [21-24]. No resistance to CPA was found in this pair of closely related lines and neither in two other pairs of closely related lines (Mogul/Borung and Cyprus/Caliph), [20].

The South Australian Research and Development Institute houses the largest collection of *M. truncatula* accessions comprising over 4000 different accessions collected from the Mediterranean basin since the 1960s. A random selection of 192 individuals constituting the *M. truncatula* core collection were analysed for genetic diversity and relationship using six simple sequence repeats (SSRs) [42]. This subset of 192 individuals has already proven to be a genetically diverse set with various sources of resistance to different isolates of necrotrophic fungal pathogens [43,44].

Here we screen a subset of 17 *M. truncatula* accessions present in the different clades of the phylogentic tree of the 192 individuals of the genetically diverse core collection to identify potential sources of CPA resistance. One accession, SA30199, showed enhanced resistance to CPA in comparison to all other *M. truncatula* accessions tested. The CPA resistance in accession SA30199 was characterised and involves antibiosis, antixenosis and tolerance. Electrical penetration graph (EPG) studies reveal that resistance is phloem based and genetic analysis identified a major quantitative trait locus (QTL) on Linkage Group 2 (LG2) explaining 39% of the antibiosis resistance phenotype. These results lay the groundwork for further molecular and biochemical elucidation of this agriculturally important trait in a well-developed plant model system.

## Results

### Screening of *M. truncatula* accessions for resistance to CPA

To assess whether there is any natural resistance to CPA in *M. truncatula*, a subset of the SARDI core collection of *M. truncatula* accessions was assayed for its performance following CPA infestation. Seventeen genetically diverse accessions representing each of the major clades in the phylogenetic tree of the SARDI core accessions generated by Ellwood and associates [42] were selected and included accessions A17 and Borung, previously identified by Gao and colleagues [20] as being susceptible to CPA. In an initial no choice glasshouse screen we found very few differences in the performances of the plants, with all accessions surviving CPA up to five weeks post infestation. Borung, SA8618 and SA9357 eventually succumbed to CPA infestation after six weeks. Typical aphid infestation phenotypes in *M. truncatula* following infestation with aphids other than CPA, such as necrotic flecks on local leaves or systemic vein chlorosis on uninfested leaves were not observed. Most accessions showed severe stunting and wilting, and damage symptoms appearing as yellowing patches or leaf chlorosis surrounding the aphid infestation sites within 1 week after infestation, with the exception of SA30199.

**Figure 1:**
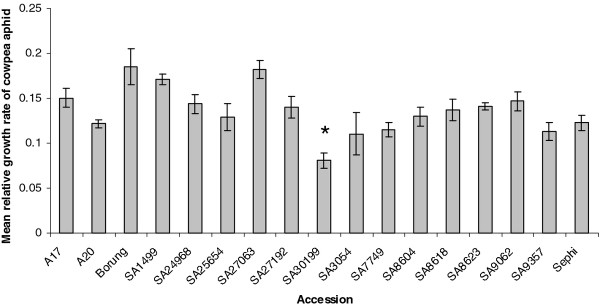
**Mean relative growth rate of cowpea aphid nymphs on 17*Medicago truncatula*accessions over four days.** Values are mean and standard error of six replicates. The * for SA30199 is to denote that the MRGR value of this accession is significantly different from the other accessions by Tukey Kramer HSD test (*P* < 0.05) with the exception of SA3054

In contrast to the plant phenotypes, there were some distinct differences in the population sizes of CPA on the different accessions with a notably lower population density on SA30199 (data not shown). In a subsequent short term infestation experiment the performance of CPA nymphs over a four day period was monitored and this reflected the plant damage and aphid densities observed at four and five weeks post infestation (Figure 1). The CPA nymph population had a significantly lower mean relative growth rate (MRGR) on SA30199 compared to all other accessions tested, with the exception of SA3054 (Tukey Kramer HSD test; *P* < 0.05). No significant differences between the accessions were found for the survivorship of CPA nymphs over this four day period (data not shown).

### Alatae prefer susceptible line Borung over resistant line SA30199

**Figure 2:**
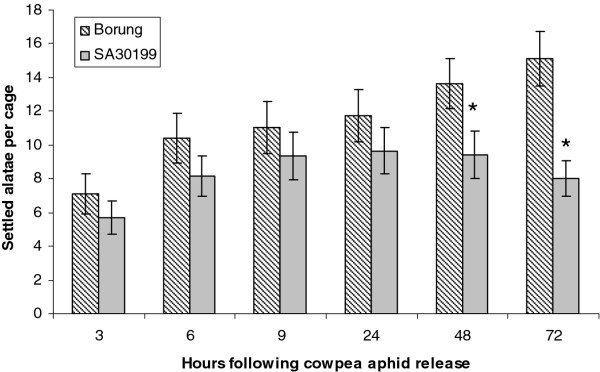
**Settling of cowpea aphid alatae in a host choice test.** Values are mean and SE of 12 biological replicates. Means labelled with * for *M. truncatula* accession SA30199 are significantly different from those for accession Borung (*P* < 0.05)

Observation of host choice by alatae (the winged, migratory morph) can reveal clues to mechanisms of aphid resistance, such as whether antixenotic (deterrent) factors are present and the speed with which they influence behavior of a foraging aphid. In the host-choice test, alatae quickly dispersed from the point of release in the centre of the cage and moved around the cage prior to settling on a plant. The average number of settled alatae increased in both Borung and SA30199 plants up to 24 h after CPA release, suggesting there was no immediate effect of an antixenotic factor (Figure 2). After 24 h, the number of aphids on SA30199 started to decline, whereas the number of alatae settled on Borung throughout the 72-h time course increased significantly (*P* < 0.01 at 48 and 72 h), suggesting a host preference by CPA.

### Resistance in SA30199 is exerted through the phloem

**Figure 3:**
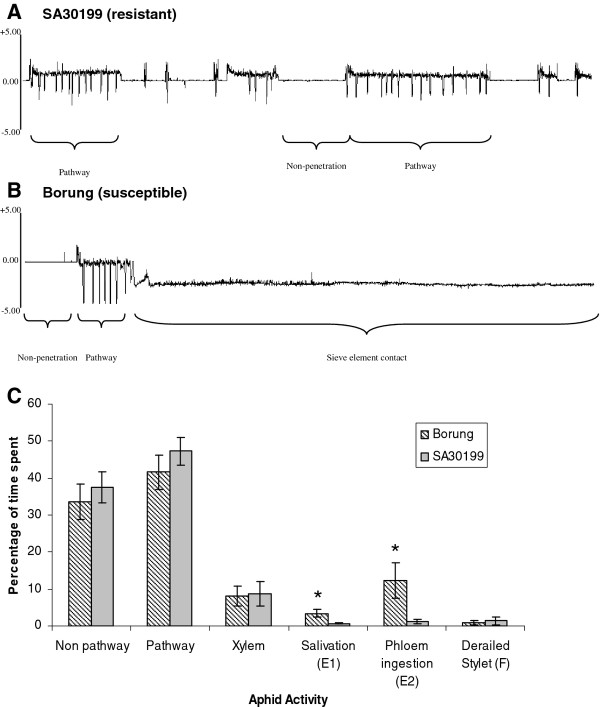
**EPG showing representative waveform patterns produced when cowpea aphid apterae feed on susceptible accession Borung (A) or on resistant accession SA30199 (B).** The horizontal axes represent a 1-h time period; the vertical axes represent voltage. Histological studies of plant-aphid interactions have correlated stylet positions in plant tissues with specific EPG waveforms [47]. “Non-penetration” indicates stylets are outside the plant. “Pathway” indicates mostly intramural probing activities between mesophyll or parenchyma cells. “Sieve element phase,” consisting primarily of sap ingestion with short periods of salivation into sieve elements, was frequently seen with plants of Borung and only rarely seen with plants of SA30199. Sharp, downward spikes, named potential drops, indicate cell puncture events by stylets, each lasting approximately 5 s. The percentage of time aphids spent in various activities on Borung or SA30199 during 9h exposure to the host plants is shown in **C**. Each value represents the mean and SE of 16 biological replicates. Means for the cultivars labeled with * are significantly different (*P* < 0.01)

The electrical penetration graph technique is a powerful method to observe in real time the locations and activities of aphid stylets during probing, including their salivation into sieve elements and passive uptake of phloem sap [45,46]. Representative traces produced by CPA probing on Borung and SA30199 are presented in Figure 3A and 3B. The proportions of time that tethered apterae spent outside the cuticle (non-probing), penetrating between cells en route to the vascular tissue (pathway phase), contacting xylem, derailed stylets or briefly puncturing cells (of unknown cell types) did not differ significantly between Borung and SA30199 (Figure 3C).

In contrast to events prior to successful puncturing of the phloem sieve element, the proportion of time spent by CPA secreting saliva in the sieve element (E1 phase) and the subsequent ingestion of phloem sap (E2 phase) were significantly reduced for CPA feeding on the resistant SA30199 plants compared to the susceptible Borung plants. Whereas the secretion of saliva on Borung plants occupied an average of 3.4% of total recorded activity, it only occupied 0.7% on SA30199 plants. Similarly, the ingestion of phloem sap on Borung plants recorded an average of 12.4%, whereas on SA30199 it averaged 1.3%. In the 16 replicates tested for each line, 87% of Borung plants had phloem sieve contact, whereas only 63% of SA30199 had phloem contact. This reduction in salivation into the sieve element and the reduction of phloem sap ingestion in contrast to all the other (pre-feeding) activities measured suggests that resistance to CPA is phloem mediated.

### Resistance in SA30199 requires an intact plant

**Figure 4:**
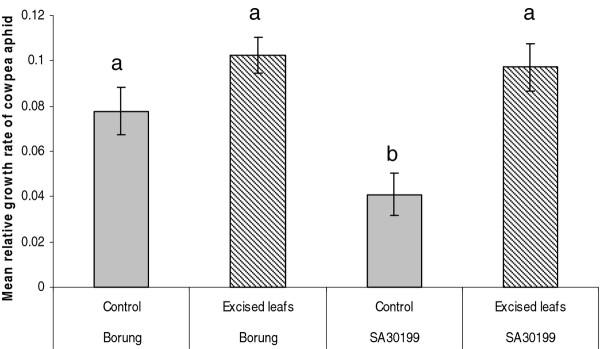
**Effects of plant genotype and shoot excision on the population growth rate of cowpea aphid.** Each value represents the mean and SE of ten biological replicates. Means labelled with the same letter are not significantly different (*P* > 0.05)

The aphid’s performance on shoots excised from the host plant in comparison with an intact plant was tested. Excision and maintenance of shoots on nutrient supplemented agar did not cause any visible wilting or other signs of damage during the four day assay. Furthermore, the aphids managed to settle on excised shoots, produce honeydew and nymphs as they would on intact plants. There was no significant difference (*P* > 0.05) in aphid survival in any of the treatments (data not shown). However, the mean relative growth rate of the aphid population on intact plants was significantly lower on the resistant SA30199 than on the susceptible Borung (*P* < 0.01; Figure 4). This resistance in SA30199 was lost on excised shoots, with aphids growing as well as they did on Borung. Excision did not significantly affect the MRGR of CPA on Borung.

### Prior infestation does not affect cowpea aphid performance on SA30199 or Borung

**Figure 5:**
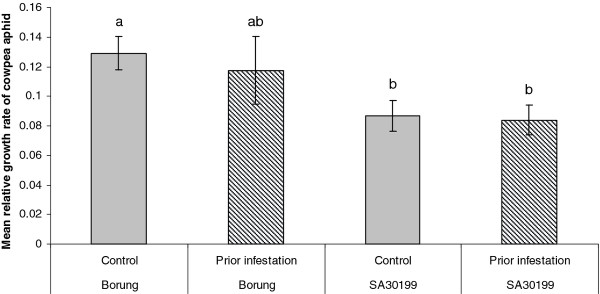
**Effects of plant prior infestation with cowpea aphid.** Values represent the mean and standard error of ten biological replicates. Means labelled with the same letter are not significantly different (*P* > 0.05)

To determine whether aphid feeding causes systemic effects on colony development, we compared aphid survival and the mean relative growth rate of the aphid colony either with or without prior infestation of Borung and SA30199. Prior infestation with CPA had no significant effect on survival relative to the no-aphid control treatment for either genotype. Similarly, no significant effect on the growth rate of the CPA population relative to the no aphid control was found (Figure 5). Therefore, we conclude that the resistance response in SA30199 does not appear to induce a systemic resistance response effective against subsequent infestations of the same aphid species.

### Resistance in SA30199 is controlled in part, by a major quantitative trait locus (QTL) on chromosome 2

**Figure 6:**
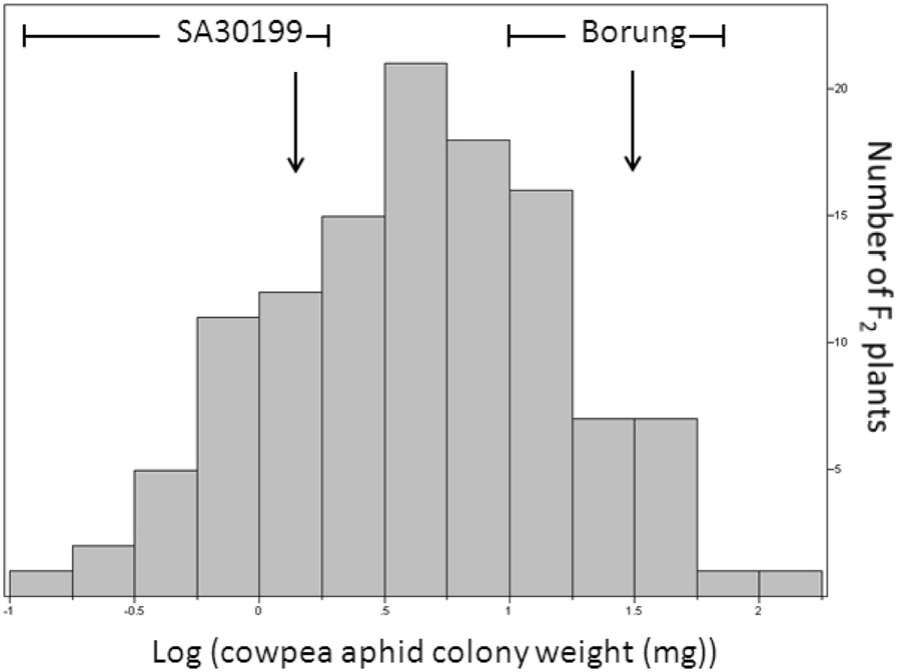
**Frequency distribution of cowpea aphid colony weight per F**_**2**_**plant 15 days after infestation with two apterous cowpea aphids.** Parental medians (downward arrow) and range (brackets) are indicated

For genetic analysis of CPA resistance, we used F_1_ hybrids and F_2_ populations from reciprocal crosses in which SA30199 was the aphid-resistant parent and Borung the aphid-susceptible parent. The F_1_ hybrids were assayed for CPA resistance and showed an intermediate resistance level between the parents SA30199 and Borung as shown in Additional file 1: Figure S1. A total of 150 F_2_ individuals (94 SA30199 x Borung individuals and 56 Borung x SA30199 individuals) were phenotyped for their response to CPA by weighing the aphid population 15 days after placing two apterous adults on each individual plant. Aphid colony weights differed markedly between parental controls (31.59 mg ± 7.53 mg SE for Borung; 1.30 ± 0.34 mg SE for SA30199; *P* < 0.01) and the distribution of the log transformed aphid weights of the F_2_ population followed a normal distribution using the Shapiro-Wilk test (as shown in Figure 6).

**Figure 7:**
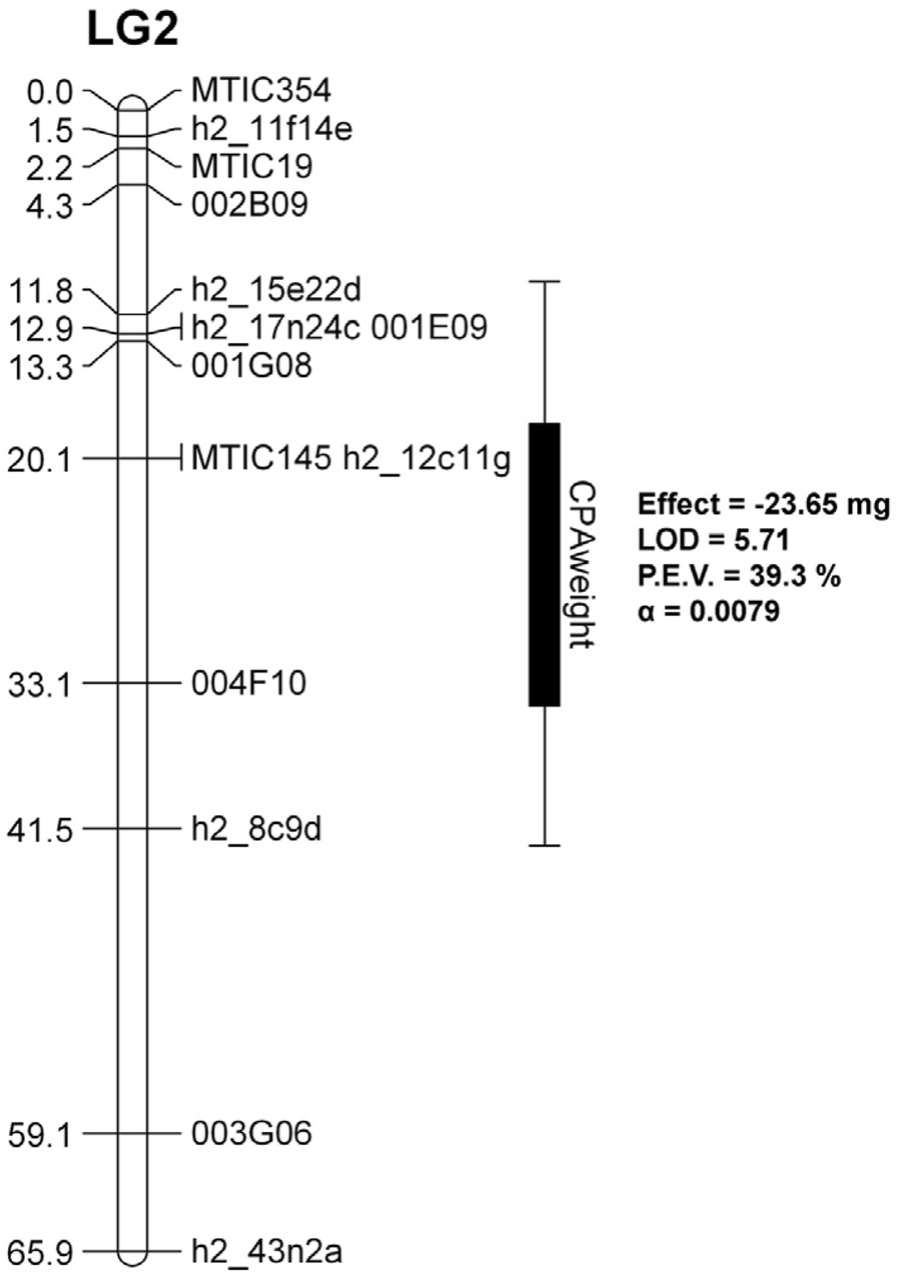
**Genetic map position of the major QTL involved in cowpea aphid antibiosis based on phenotype data for the F**_**2**_**individuals of the SA30199 x Borung population.** Interval distances are listed in centiMorgans. The genomic location of the QTL is depicted on the right of linkage group 2 (LG2), with standard deviations depicted by lines either side

To identify the region controlling CPA antibiosis in SA30199, QTL analysis was performed using the CPA colony fresh weight data. This analysis identified a highly significant locus with a LOD score of 5.71 explaining 39.0% of the phenotype for CPA fresh weight (Figure 7) on chromosome 2 (LG2). No other QTLs for CPA colony fresh weight were identified.

## Discussion

The use of *M. truncatula* as a model system to characterize and determine the genetic basis of aphid resistance provides an opportunity to improve resistance to sap-sucking insects in agriculturally important legume crops, where aphids can be significant problems [48]. The use of this system has already lead to the identification of single dominant and semi-dominant resistance genes for BGA, PA and SAA [21-25]. Besides these resistance genes, quantitative trait loci controlling different aspects of aphid resistance (e.g. antibiosis and tolerance) have also been identified in *M. truncatula* for BGA and PA [49]. While no resistance to CPA has previously been identified in *M. truncatula,* only three pairs of closely-related lines of *M. truncatula* were assayed and no significant differences were identified following CPA infestation [20]. The present study screened a subset of the SARDI core collection of *M. truncatula* accessions, that span the genetic diversity in this collection, for resistance to CPA. One accession, SA30199, was found to have an increased resistance to CPA compared to all other *M. truncatula* accessions tested with the exception of SA3054 (Figure 1). In initial no choice glasshouse screens, no visual differences were found in plant performance, with all *M. truncatula* accessions showing degrees of stunting and wilting, and no obvious other macroscopic phenotypes such as local necrotic flecks as observed following bluegreen aphid or pea aphid infestation in some *M. truncatula* accessions [21,23] or systemic vein chlorosis as seen following spotted alfalfa aphid infestation in susceptible *M. truncatula* accessions [24]. However, distinct differences in the population sizes of CPA on the different accessions with a notable lower population density on SA30199 was observed and confirmed in a subsequent short term experiment, where CPA had a significantly lower MRGR on *M. truncatula* SA30199 compared to all other *M. truncatula* accessions tested (Figure 1).

Host selection by alatae is regarded as the first stage of colonisation and therefore plays a major role in aphid establishment in the field. In repeated host choice experiments with alatae of CPA, we found that the alatae preferentially settled on the susceptible accession Borung compared to the resistant SA30199 from 48h after release (Figure 2). This is in contrast to the response of BGA in similar experiments where a clear preference for the susceptible line A17 compared to the resistant line Jester was visible within 6 h of release [23]. However, the response of PA in a similar experiment using the lines Jester and A17 showed a similar result in host preference as seen for CPA with the settling preference only becoming apparent after 48 from release [21], suggesting antixenosis is involved in the response and is likely derived from the inability to establish a successful feeding site.

In Electrical Penetration Graph (EPG) studies using adult CPAs, a reduction in salivation into the sieve element and a reduction of phloem sap ingestion on resistant SA30199 plants compared to susceptible Borung plants were identified. These significant differences, in contrast to the all other (pre-feeding) activities measured, suggest that resistance to CPA is phloem mediated. This combined with the host choice results also suggest the antixenotic effect of resistance, observed in the choice test, may be derived from the inhibition of sap uptake rather than cues from volatile compounds or surface waxes from SA30199. This is supported by the proportion of time that tethered apterae spent outside the cuticle, penetrating between cells en route to the vascular tissue, contacting xylem, derailed stylets or briefly puncturing cells, which did not differ significantly between Borung and SA30199 (Figure 3C). The similarities between the behaviour of the aphids for these activities suggest that neither surface features (e.g. epicuticular waxes or trichomes) nor cell wall properties play a role in the resistance mechanism of SA30199. Feeding behaviour of CPA was also examined in narrow-leafed lupin, where CPA spend significantly more time in non-probing and stylet pathway activities and significantly less time in the phloem sieve element on the resistant cultivar Kalya compared to the susceptible cultivar Tallerack [50]. Similarly, resistance to CPA in cowpea resistant cultivar ICV-12 compared to susceptible cultivar ICV-1 showed a reduction in the phloem sieve element phase [29]. In all three cases a phloem-based deterrence therefore plays the major role in CPA resistance. Similar experiments conducted in *M. truncatula* lines resistant and susceptible to BGA and PA revealed significant reductions in phloem feeding on the resistant lines and therefore all the identified aphid resistance in *M. truncatula* to date appears to be exerted through the phloem [21,23].

If CPA resistance in SA30199 is based on phloem properties, the causal factor may be produced locally. This could be achieved by either a physical blockage of sap uptake at the feeding site through rapid polymerization and deposition of macromolecules such as callose or phloem proteins or by biosynthesis of resistance factors in the vicinity of aphid feeding sites. However, our finding that shoot excision eliminates CPA resistance in *M. truncatula* SA30199 (Figure 4) raises the possibility that a resistance factor(s) imported from the root or a root to shoot signal could be required to retain CPA resistance. The loss of phloem-based resistance to CPA on excised shoots of SA30199 (Figure 4) has also been observed in the resistance to BGA and PA in the *M. truncatula* accession Jester [21,23]. Reciprocal grafting experiments between SA30199 and Borung will be necessary to confirm the hypothesis that a resistance factor(s) is imported to the feeding site.

Pre-infestation (with CPA) had no significant effect on subsequent CPA feeding and the growth rate of the CPA population (Figure 5), which is similar to the observations made by Gao and colleagues [21] who characterised PA resistance in *M. truncatula.* However, the CPA and PA findings contrast with BGA resistance characterised in *M. truncatula*, where prior infestation with BGA on resistant lines showed a significant reduction in the population growth rate of a subsequent BGA infestation [23].

The biology of CPA resistance in *M. truncatula* SA30199 shares similarity with resistance to other aphid species in *M. truncatula* as it involves a combination of antibiosis, antixenosis and tolerance and resistance is phloem based. All the major aphid resistance loci in *M. truncatula* have been mapped to chromosome 3 in regions rich in open reading frames (ORFs) encoding nucleotide-binding-sites leucine-rich-repeats (NBS-LRRs). The three cloned aphid resistance genes identified to date; the *Mi* gene to potato aphid (*Macrosiphum euphorbiae*) in potato, the *Vat* gene to cotton-melon aphid (*Aphis gossypii*) in melon and the resistance gene to lettuce root aphid (*Pemphigus bursarius*) in lettuce belong to the CC-NBS-LRR class of resistance genes [14,51,52]. Furthermore, the *AIN* locus, which explains 88% of the antibiosis resistance in *M. truncatula* A17 to BGA and 23% of the antibiosis effect to PA, resides in a cluster of paralogous NBS-LRR genes [25]. The major CPA antibiosis QTL was identified on the short arm of chromosome 2. The homologous region spanning the QTL on chromosome 2 in the reference genome of A17 was queried for the presence of NBS-LRR domains. This identified 9 Toll-like Interleukin 1 Receptor (TIR) and 5 CC-NBS-LRR domains respectively. This region is thus not as dense in, but does contain NBS-LRR domains. Further fine-mapping and subsequent cloning of this locus will elucidate whether the locus controlling CPA antibiosis belongs to the NBS-LRR class of aphid resistance genes.

## Conclusion

With the frequent breakdown of single dominant *R* genes in our extensively monocultured agricultural systems, it is important to pyramid multiple resistance genes into crops with major *R* genes to help inhibit the occurrence of new virulent biotypes. CPA is the most important insect pest of cowpea and also causes significant yield losses in other legume crops including alfalfa, beans, chickpea, lentils, lupins and peanuts [29,35-37,40,53]. Here we have identified the first strong antibiosis resistance in *M. truncatula* against CPA, an economically important legume pest. The identified locus will facilitate marker assisted breeding of *M. truncatula* for increased resistance to CPA and potentially other closely related Medicago species such as alfalfa. Using synteny with soybean (*Glycine max*) and *M. truncatula*, candidate resistance genes have been identified within mapped QTL intervals in cowpea for *Macrophomina phaseolina* resistance [30] and synteny studies could also help identify homologous genes to the CPA resistance gene (s) identified in *M. truncatula*. The identification and characterization of this novel resistance locus to CPA in a model plant further adds to the potential *M. truncatula* has for studying plant defense against sap-sucking insects. It has proven difficult to introduce resistance from wild relatives into certain cultivated legume species due to sexual incompatibilities and high degrees of autogamy. The *M. truncatula*-CPA system would allow us to identify key regulators and genes important on the plant side of the interaction to give effective resistance. The homologous genes in cowpea, chickpea and other legume crops could then serve as targets for over-expression to enhance resistance to CPA.

In the last decade, increasing efforts in the area of aphid genomics have been made and include the sequencing of the first aphid genome [54], the availability of numerous EST and transcriptome sequence datasets (see [55]) and RNA interference approaches to knock down aphid genes [56,57]. Using these resources and the advances in next generation sequencing technology would also allow us to further the development of novel strategies to identify key CPA genes involved in host manipulation to establish a feeding site and/or effectors recognized by the resistance locus. Using RNAi-based approaches in legume crops targeting these essential aphid genes could lead to effective durable resistance to this destructive pest.

## Methods

### Plants and aphids

*M. truncatula* seeds were obtained from the Genetic Resource Centre, SARDI (South Australian Research and Development Institute, Adelaide, South Australia). To ensure even germination, seeds were scarified using sandpaper and transferred to a Petri dish lined with blotting paper, and irrigated with sterile water. The seeds were kept at room temperature for 48 h, and germinated seedlings were planted in Arabidopsis mix (Richgro Garden Products, Jandakot, Western Australia 6164). The plant growth conditions were as described previously by Klingler et al. [25]. Plants were grown in individual 0.45 L pots in a temperature controlled glasshouse.

The cowpea aphids used in this study were from an asexual, parthenogenetic strain collected in Western Australia, derived from single-aphid isolates, and maintained in the laboratory as described by Gao et al. [20]. Aphids were transferred to experimental plants with a fine paintbrush.

### Aphid performance on caged leaves

CPA was infested on seventeen genetically diverse accessions from each of the major clades in the phylogenetic tree by Ellwood et al. [42]. The survival and growth rate of CPA were measured after four days on individual plants of each *M. truncatula* accession with six replicates for each accession. Two weeks after sowing, a cohort of eight pre-weighed, early-instar nymphs was placed on a trifoliate leaf of each plant and caged. Four days after the infestation, the number and weight of surviving aphids on each plant were recorded. The mean relative growth rate (MRGR) of surviving nymphs was calculated as the difference between the logarithms of the initial mean weight of aphids placed on the plant (W_orig_) and the final, mean weight of living aphids removed from the plant (W_sur_) [20]: MRGR = (log W_sur_ - log W_orig_)/number of days. The proportion of aphids that survived and MRGR were compared using the Tukey-Kramer Honestly Significant Difference test with the JMP-IN 5.1 software (SAS Institute, Cary, NC).

### Aphid performance on caged plants

In a test of CPA colony growth on genotypes SA30199, Borung and their F_1_ progeny_,_ twelve individual three-week-old plants were each infested with two apterous adults of CPA in a growth chamber. Each individual plant was subsequently covered with a whole plant cage made from a clear plastic bottle modified with a cut-off base and large mesh-covered ventilation holes. Fifteen days after infestation the bottles were removed and the aphids on each plant were gently brushed off and immediately weighed. Means of the aphid fresh weight were subjected to one-way ANOVA and compared using the Tukey-Kramer Honestly Significant Difference test with the JMP-IN 5.1 software. This method was selected to subsequently phenotype an F_2_ population derived between SA30119 and Borung.

### Host selection behaviour

Twenty plants each of SA30199 and Borung were grown in separate 0.45L pots in a temperature controlled glasshouse. Three weeks after sowing, two plants of Borung and two plants of SA30199 were placed in each of ten insect-proof cages (38 cm length x 28 cm width x 46 cm height) covered with fine, light-transmitting mesh on the top and on three sides, and a sliding Perspex cover on the remaining side. Two plants of each genotype were randomly placed in the cage so that one plant occupied each of the four corners. Pots were spaced so that no leaves touched other plants. A 5 cm Petri dish was placed in the centre of the cage, suspended at a height of approximately 10 cm above the soil level of each pot. Fifty CPA alatae were placed on the platform in each cage and allowed to choose host plants on which to feed and reproduce over the next 72 h. Settling of aphids on each plant was observed at 3, 6, 9, 24, 48 and 72 h after release. The significance of the difference in the settling of aphids (pooled data) between SA30199 and Borung and among time points was analysed with two-way ANOVA (genotype x time points) using JMP-IN 5.1 software.

### Aphid feeding behaviour

The feeding behaviour of CPA on SA30199 and Borung was studied using the direct-current electrical penetration graph (EPG) technique [45] as described in Klingler et al. [23] with modifications. Plants were grown with 16 h light (22°C)/8 h dark (20°C) under metal halide and incandescent lamps producing 300 mE m22 s21. When plants were 3 to 4 weeks old, a single apterous adult CPA was placed on a single trifoliate leaf and the feeding behaviour was monitored. Fifteen biological replicates were included for each SA30199 and Borung genotype. A six channel amplifier simultaneously recorded six individual aphids on separate plants, three SA30199 and three Borung per day for five days. Waveform patterns in this study were scored according to categories described by Tjallingii and Esch [46]: non-penetration; pooled pathway phase activities; salivary secretion into sieve elements; phloem sap ingestion; xylem ingestion; and cell puncture events of several seconds’ duration (referred to as potential drop). The mean proportion of time spent in each behaviour on each plant of the two cultivars was analysed by one-way ANOVA using JMP-IN 5.1 software.

### Aphid development on intact plants and excised leaves

The survival and growth rate of CPAs were measured after 4 days on individual intact plants or excised shoots of each *M. truncatula* accession, SA30199 or Borung, with ten replicates for each treatment. The plant growth, leaf excision and culture, and measurement of the survival and growth rate have been previously described by Klingler et al.*,*[23] and Gao et al.*,*[20], with some modifications. For the excised leaves, a stem tip with one node was excised from each plant of Borung or SA30199 three weeks after planting and inserted into agar supplemented with soluble fertilizer in an inverted 90-mm-diameter Petri dish. A cohort of eight first- or second-instar nymphs was placed on each of the intact plants or excised leaves of SA30199 or Borung. The proportion of aphids that survived and aphid MRGR were analysed by two-way ANOVA (genotype: SA30199 and Borung; treatment: leaf excision and intact plant) using JMP-IN 5.1.

### Aphid performance on pre-infested plants

To assess the effect of pre-infestation with CPA on the performance of CPA on SA30199 and Borung, aphid survival and growth were measured after 4 days on pre-infested and control plants of each cultivar using cohorts of 10 pre-weighed, early-instar CPA nymphs as described by Klingler et al. [23]. Plants were grown in individual 0.45 L pots in a growth chamber. Four weeks after sowing, a linen mesh cage (35 x 200 mm) was placed on a single trifoliate leaf of each plant with a wooden stake supporting the stem and cage. The cage was placed on either the fourth or fifth trifoliate leaf to emerge on the primary stem of each plant. Twenty CPA adults were placed inside the cage and none in the uninfested caged control. Aphids had access to the stem, a single trifoliate leaf, and its petiole. At the end of the 2 day pre-infestation treatment, a mesh cage was placed on the next trifoliate leaf distal to (younger than) the original caged leaf on the same stem. A cohort of 10 pre-weighed, early-instar nymphs of CPA was placed inside this second cage, whereas the original aphids remained in their cage on the other leaf. Four days after the second infestation, the number and weight of surviving aphids in the second cage were recorded. The MRGR of surviving nymphs was calculated as described above. The proportion of aphids that survived and MRGR were compared by two-way ANOVA (genotype: SA30199 and Borung; treatment: pre-infestation and no pre-infestation) and compared by the LSD test at a 5% significance level using JMP-IN 5.1.

### Genetic analysis of cowpea aphid resistance

Parental *M. truncatula* accessions were identified from the resistance phenotype screens described above as being most resistant (SA30199) and most susceptible (Borung) and reciprocal crosses between SA30199 and Borung were obtained by a manual crossing procedure described by Thoquet et al.*,*[58] and their nature confirmed by polymerase chain reaction (PCR) using DNA markers polymorphic between the parental lines. One hundred and fifty F_2_ individuals were screened for their resistance to CPA by placing two apterous adults on a three week old individual F_2_ plant. Each individual plant was covered as described above and after fifteen days the aphids were gently brushed off and immediately weighed.

DNA was isolated from the parental lines and the 192 F_2_ individuals (as described in Kamphuis et al., [44] and genotyped using 100 polymorphic primer pairs out of 204 primer pairs tested for polymorphisms between the parents. The primer pairs were designed by the *Medicago* genome sequencing project (http://www.medicago.org/genome/) and the polymorphic markers were genotyped using the high-throughput multiplex ready technology (MRT; [59]) optimized for *M. truncatula* as described by Guo et al. [49]. A genetic map was constructed using the Multipoint version 1.2 software (http://www.multiqtl.com; Institute of Evolution, Haifa University, Haifa, Israel) with the parameters as described by Kamphuis et al. [60].

QTL analysis was performed with MultiQTL v2.5 by applying a general interval mapping and marker restoration method as described by Kamphuis et al. [44] with the evaluation of the hypotheses that a single locus or two linked loci have an effect on resistance to CPA. Firstly, 5000 permutation tests were performed on the hypothesis that one locus on a chromosome has an effect on the disease resistance (H_1_) versus the null hypothesis (H_0_) that the locus has no effect on the disease resistance. Secondly, 3000 permutation tests were run on the hypothesis that a single locus has an effect on CPA resistance versus two linked loci. The model with the highest LOD score was fitted to the QTL and when the models did not differ significantly the simpler model was chosen ('one locus-one trait'). Five thousand bootstrap samples were run to assess the estimates and the standard deviation (SD) of the main parameters: locus effect, its chromosomal position, its LOD score and the proportion of explained variability (PEV).

## Abbreviations

CPA, Cowpea aphid; EPG, Electrical penetration graph; LG, Linkage group; MRGR, Mean relative growth rate; QTL, Quantitative trait locus.

## Competing interests

The authors declare they have no competing interests.

## Authors’ contributions

LGK, LLG and KBS designed the experiments. LGK conducted the experiments. LGK drafted the manuscript with help from LLG and KBS and all authors discussed results, commented on and approved the final manuscript.

## Supplementary Material

Additional file 1 **Figure S1 **Mean relative growth rate of cowpea aphid on the parents SA30199 and Borung and the F_1_ individuals over four days. Values are the mean and standard error of 11 biological replicates for the parents and the F_1_ individuals (nine and two for the SA30199 x Borung cross and Borung x SA30199 cross, respectively). Means with a different letter are significantly different by Tukey Kramer HSD test (*P* < 0.05) (DOCX 25 kb) Click here for file
