# Supplementary material for: Identification and characterization of resistance to cowpea aphid (Aphis craccivora Koch) in Medicago truncatula
Source: BMC Plant Biol. 2012 Jul 4;12:101. doi: 10.1186/1471-2229-12-101 (PMC3464659; doi:10.1186/1471-2229-12-101)
Supplement: Additional file 1 — Figure S1 Mean relative growth rate of cowpea aphid on the parents SA30199 and Borung and the F1 individuals over four days. Values are the mean and standard error of 11 biological replicates for the parents and the F1 individuals (nine and two for the SA30199 x Borung cross and Borung x SA30199 cross, respectively). Means with a different letter are significantly different by Tukey Kramer HSD test (P < 0.05) (DOCX 25 kb) [file 1471-2229-12-101-S1.docx]

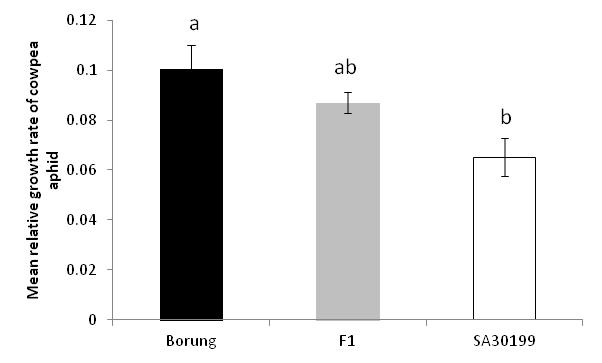


Figure S1. Mean relative growth rate of cowpea aphid on the parents SA30199 and Borung and the F_1_ individuals over four days. Values are the mean and standard error of 11 biological replicates for the parents and the F_1_ individuals (nine and two for the SA30199 x Borung cross and Borung x SA30199 cross, respectively). Means with a different letter are significantly different by Tukey Kramer HSD test (P < 0.05).
